# Neuropathic Pain in Neurologic Disorders: A Narrative Review

**DOI:** 10.7759/cureus.22419

**Published:** 2022-02-20

**Authors:** Dimos-Dimitrios Mitsikostas, Eleni Moka, Enrique Orrillo, Caterina Aurilio, Athina Vadalouca, Antonella Paladini, Giustino Varrassi

**Affiliations:** 1 First Department of Neurology, Eginition Hospital, National and Kapodistrian University of Athens, Athens, GRC; 2 Department of Anesthesiology, Creta Interclinic Hospital, Herakleion, GRC; 3 Pain Medicine, Asociacion Peruana para el Estudio del Dolor, Lima, PER; 4 Department of Anaesthesiology, University of Napoli, Napoli, ITA; 5 Pain and Palliative Care Center, Athens Medical Hospital, Athens, GRC; 6 Department of Life, Health and Environmental Sciences (MESVA), University of L'Aquila, L'Aquila, ITA; 7 Pain Management, Paolo Procacci Foundation, Rome, ITA

**Keywords:** pain, neuropathic pain, neurological disorders, long covid, multiple sclerosis, post-stroke pain

## Abstract

Neuropathic pain is defined as a painful condition caused by neurological lesions or diseases. Sometimes, neurological disorders may also be associated with neuropathic pain, which can be challenging to manage. For example, multiple sclerosis (MS) may cause chronic centralized painful symptoms due to nerve damage. Other chronic neuropathic pain syndromes may occur in the form of post-stroke pain, spinal cord injury pain, and other central pain syndromes. Chronic neuropathic pain is associated with dysfunction, disability, depression, disturbed sleep, and reduced quality of life. Early diagnosis may help improve outcomes, and pain control can be an important factor in restoring function. There are more than 100 different types of peripheral neuropathy and those involving sensory neurons can provoke painful symptoms. Accurate diagnosis of peripheral neuropathy is essential for pain control. Further examples are represented by gluten neuropathy, which is an extraintestinal manifestation of gluten sensitivity and presents as a form of peripheral neuropathy; in these unusual cases, neuropathy may be managed with diet. Neuropathic pain has been linked to CoronaVirus Disease (COVID) infection both during acute infection and as a post-viral syndrome known as long COVID. In this last case, neuropathic pain relates to the host’s response to the virus. However, neuropathic pain may occur after any critical illness and has been observed as part of a syndrome following intensive care unit hospitalization.

## Introduction and background

Neuropathic pain affects about 3-17% of the general population [1] and is characterized by positive (extra sensation perceived by the patient, such as pain, paresthesia, numbness, and tingling) and negative aspects (loss of functions such as sensory, motor, and cognitive deficits) [2]. Neuropathy may be part of mixed pain syndromes and can be a component in chronic pain [2]. Usual analgesics efficacious to treat nociceptive pain may be ineffective in neuropathic pain.

Neuropathic pain has been defined as a consequence of certain endocrine dysfunctions (e.g., painful peripheral diabetic neuropathy), viral infections (e.g., postherpetic neuralgia), trauma (e.g., spinal cord injury), and as a treatment-emergent effect (e.g., chemotherapy-induced peripheral neuropathy), among others. Neuropathic pain has also been associated with cancer or neurologic disorders [e.g., multiple sclerosis (MS), stroke, centralized pain syndromes]. Rapid diagnosis helps ensure optimal outcomes, and neuropathic control in patients with neurological conditions relies on symptomatic relief usually through multimodal pharmacologic therapy. As a rule of thumb, first-line approaches include gabapentinoids, tricyclic antidepressants, and serotonin-norepinephrine reuptake inhibitors (SNRIs), however, opioids, topical lidocaine or capsaicin, and even botulinum toxin may be considered as second- or third-line therapies [1]. New treatments are under consideration, as the mechanisms underlying neuropathic pain are better elucidated.

## Review

This review is originated from the presentations made by the authors in a congress. The material has been synthesized and updated searching on PubMed, Scopus, and WoS, especially focusing on specific neurological disorders (e.g., MS) and their connection with neuropathic pain. The aim is to provide the readers with updated scientific material for their everyday clinical practice, both on specific neurological disorders and on their connection with neuropathic pain.

Multiple sclerosis

MS is a chronic and potentially disabling disease of the central nervous system in which the body’s immune system attacks the myelin sheath around certain nerve fibers. It is a complex disease with several known phenotypes [3]. It can result in functional motor and sensory deficits caused by immune-mediated inflammatory processes, demyelination, and axonal damage [4]. This damage is related to several idiopathic inflammatory-demyelinating diseases of the central nervous system [5].

While MS involves demyelination in the central nervous system, disease severity, symptomatology, and disability do not correlate well with the degree of demyelination [6]. Earlier it was thought that MS was mediated primarily by pro-inflammatory T-cells that were activated by the autoimmune system. However, it is now believed that β-cells, dendritic cells, and monocytes also play the main role [7]. Oligodendrocytes produce myelin to metabolically support the nerve axons [8]. In animal studies, it was found that new oligodendrocytes form to replace damaged myelin, but this task is not performed by existing oligodendrocytes. It is not clear if, and to what extent, this may occur in humans [9].

The prevalence of MS ranges from 2 per 100,000 in Japan and Sub-Saharan Africa to 100 per 100,000 in North America and Northern Europe [10]. MS reduces life expectancy by six to seven years, although a study found no differences in mortality in the first 20 years of the disease [11]. Relapsing-remitting MS, the most frequently encountered form of the disease, is more prevalent in women than in men [11]. In the United States, the highest prevalence of MS occurs among people between the ages of 45 and 49 years [12]. MS can be heritable but genetic markers have not been completely elucidated [11]. A study from Spain reported that the mean age at the first symptom of MS was 32.2 years, the average delay to diagnosis was 3.1 years, and 71.2% of patients had relapsing-remitting MS [13]. Cold countries seem to have a higher prevalence of MS [14] but there is no bright line dividing North and South in this respect [15]. Globally, about 2.5 million people have some form of MS [16].

The clinical course of MS can be challenging to describe because of the range of different symptoms, their relative severity, and outcomes [17]. The main phenotypes of MS were at first defined only clinically: relapsing-remitting MS, primary or secondary progressive MS, and progressive-relapsing MS. When these original phenotypes were reconsidered, the radiologically isolated syndrome and the clinically isolated syndrome were added to improve the descriptors by incorporating activity and disease progression into the phenotype [18]. The inflammatory response in MS is transient. The demyelination and remyelination phases lead to periods of relapse and remission, but over time, remyelination effects are not durable. Relapsing-remitting MS is clinically characterized by episodes of acute exacerbation of neurologic symptoms punctuated by periods of partial recovery, but with no apparent progression of the disorder. However, primary progressive MS is characterized by a steady, progressive loss of neurologic function with no distinct episodes of remission. Secondary progressive MS likewise manifests as a steady loss of neurologic function but maybe punctuated by episodic remission. In progressive forms of MS, microglial activation and the widespread neurological damage it promotes result, over time, in neurodegeneration, prominent symptoms, disability, and concomitant neuropathic pain [17].

The radiologically isolated syndrome is diagnosed when incidentally identified abnormalities in an imaging study suggest demyelination before there are clinical signs or symptoms of MS [19]. The clinically isolated syndrome occurs when a patient exhibits clinical signs or symptoms suggesting MS but does not meet the diagnostic criteria for MS. These symptoms are usually acute, monofocal clinical events, such as abnormalities of the optic nerve [20]. These syndromes are emerging as important for MS investigations, even if they are arguably not true phenotypes.

Approximately 29-86% of MS patients complain about pain, and central neuropathic pain is present in the majority of cases [21]. About 80% of MS patients develop spasticity as well [21]. Among the most frequently reported painful symptoms of MS are pain in the extremities, trigeminal neuralgia, low back pain (LBP), and headache, none of which reliably respond to conventional analgesic therapy [21]. Painful symptoms are more likely to be reported with disease progression than at its outset [22]. Of course, pain may occur at any point in the disease and, in some cases, reported pain is secondary to spasticity, mood disorders, and fatigue [23]. While MS patients report low levels of satisfaction with analgesic therapy, about 30% of all drugs prescribed to MS patients are prescribed to help manage pain [23]. Patient self-reports of pain are important to consider in MS because the incidence and severity of pain in MS do not necessarily correlate with the extent or severity of the underlying disease [24].

Although headache is a common symptom of MS, its prevalence and type vary. Migraine is frequently observed, but it remains unclear whether migraine is merely associated with MS or is a true comorbidity [25]. In a study of MS patients (*n*=137), the lifetime prevalence of headache was 57.7%, with 31.9% having tension-type headache and 25.0% migraine. Migraine has been significantly correlated to relapsing-remitting MS but causal mechanisms have not been identified [26]. In some cases, patients had pre-existing headaches that either continued or were exacerbated by MS [27]. In a study comparing MS patients to the general population, the relative frequency of migraine headaches among MS patients was three-fold higher than for the general population both for women (55.7% vs. 17.1%, prevalence ratio 3.26, *p*<0.001) and men (18.4% vs. 5.6%, prevalence ratio 3.29, *p*<0.001), although migraines occur more frequently in women [28]. In a study of 18,955 MS patients, migraine prevalence was 7%, compared to 2.8% in the control group [29]. The “coexistence” of migraine with MS is well documented, but a causal link is missing [30]. A meta-analysis found that the prevalence of migraine in MS patients was inconsistent, but varied by geography [31]. It has been suggested that the altered sensory perceptions migraineurs experience during headache might cause them to experience typical MS symptoms of paresthesia or pain as migraine [32]. The prevalence of all types of headaches is on the increase in the general population and sedentary lifestyle, unhealthful diet, stress, poor posture, and increased use of digital media (“screen time”) have been implicated in what has been termed “the 21st-century headache” [33]. Thus, it is difficult to distinguish such headaches from those caused by MS.

Nevertheless, pain is prevalent in the MS population, even during the early stage of the disease, and it is challenging to treat, partly because its underlying mechanisms are unclear. There is no unequivocal guidance with respect to how MS-associated pain should be managed. Nonpharmacologic treatments such as transcutaneous electric nerve stimulation, psychological counseling, transcranial direct stimulation, hydrotherapy, and reflexology have all been researched, but there is limited evidence to recommend any of these treatments [34]. A randomized controlled clinical study of MS patients assigned to do yoga or aerobic exercise (*n*=90) found that at 12 weeks, both yoga and aerobic exercise decreased MS symptoms, reduced days lost from work, and increased the patient’s efficiency in daily life [35]. Individualized exercise programs can be beneficial for appropriate MS patients [36].

Multimodal pharmacologic regimens have been recommended that may include opioids, nonsteroidal anti-inflammatory drugs (NSAIDs), anticonvulsants, and antidepressants but such treatments are not always effective in the MS population and clinicians must also consider potential side effects [21]. New treatment modalities, such as cannabis-based treatments and neuromodulation, are being explored [21].

Spasticity is prevalent in MS patients and may cause pain, fatigue, functional deficits, and disability [37]. While spasticity may affect many different muscles and can wax and wane with temperature, activity levels, and other factors, lower extremities are most commonly affected [37]. MS-related spasticity may include muscle spasms, myoclonus, clonus, co-contraction, stiffness, resistance to passive movement, lost coordination and dexterity, slowed movement, and weakness [37].

The estimated number of MS patients around the world is increasing and expected to continue to increase, with the most striking increases in Africa (59% increase in prevalence from 2013 to 2020), the Americas (87%), and Southeast Asia (58%) [38]. As MS is an incurable, lifelong condition that increases the risk of neurological dysfunction, it is crucial that it is better understood with respect to safe and effective treatment regimens.

Painful peripheral neuropathy

There are several different types of peripheral neuropathy, and those involving the sensory neurons can be associated with moderate to severe pain. Many forms of neuropathy involve all three of the main types of nerves to some extent: sensory, motor, and autonomic nerves. Neuropathy may occur with damage to small- and/or large-fiber myelinated or unmyelinated nerves. Peripheral neuropathy typically involves small-fiber nerves and can provoke classical neuropathic painful symptoms perceived as burning, “electrical,” or shooting pain as well as allodynia [39].

Peripheral neuropathies are often polyneuropathies, involving multiple nerves. They may be described by the type of nerves affected (sensory, motor, autonomic), the site of nerve injury (distal or proximal), the nerve component affected (demyelinating or axonal), etiology (e.g., diabetic neuropathy), or pattern (symmetric or asymmetric) [40]. Length-dependent neuropathy affects the longer fibers, as a result of which the more distal areas of the body are affected first. Described as a “dying back” phenomenon, length-dependent neuropathy typically affects toes and fingers first and is a form of sensory neuropathy. In contrast, asymmetrical neuropathy can be purely sensory with a patchy distribution and no discernible pattern; asymmetrical neuropathy is common in cancer patients but may occur in other patients as well. Asymmetrical sensorimotor neuropathy involves multiple motor neurons and can lead to vasculitis, and it is relatively rare.

About two-thirds of all patients with peripheral neuropathy have painful symptoms, which can be challenging to manage effectively [41]. The diagnosis and treatment of peripheral neuropathy can be complicated further by the fact that some patients are asymptomatic or have only diffuse symptoms [42]. Peripheral neuropathy is a common disorder, and the large subpopulations who are geriatric, obese, and/or have diabetes are at elevated risk [43]. The prevalence of peripheral neuropathy is 2.4% in the general population. It increases to 8% in individuals above 55 years [44,45]. It occurs in 12.1% of obese but normoglycemic patients and in 40.8% of obese individuals with diabetes [46].

Length-dependent peripheral neuropathy is symmetric and begins at the termini of the longest nerves, that is, in the feet and toes. Typically, a loss of sensation, numbness, or unusual sensory symptoms, such as tingling, burning, “pins and needles,” or electrical sensations, occurs antecedent to motor weakness [47]. Pain locations are symmetric and gradually move upward, with symptoms in the hands manifesting around the same time as symptoms occur in the knee and leg. There are no deficits in proprioception until the condition advances [47]. Polyradiculoneuropathy and multifocal neuropathy may arise in the presence of cancer, infections, vasculitis, inflammation, or other conditions [47].

The diagnosis of peripheral neuropathy should include a detailed patient and family history, physical examination, serologic testing, and an assessment of the patient’s comorbidities. In most cases, a length-dependent peripheral neuropathy can be diagnosed, but in 20% of cases, the etiology of the peripheral neuropathy remains unknown [48]. It is important to determine whether the neuropathy is axonal or involves demyelinated fibers, as treatment courses differ. The underlying cause of the neuropathy, if known, can be crucial to finding an effective treatment. For instance, peripheral neuropathy may be caused by cancer or chemotherapy, a viral infection, or vasculitis. Axonal peripheral neuropathy is caused by inflammation, infection, ischemia, metabolic disruptions, or may be genetic. A range of conditions is associated with neuropathy: diabetes, vitamin B-12 deficiency, renal dysfunction, hypothyroidism, and CoronaVirus Disease (COVID), among others. Neuropathy may also arise from long-term excessive alcohol consumption, certain prescription drugs, or the use of other neurotoxic agents [47,49].

Diagnostic tests may involve electromyography (EMG) and nerve conduction studies. EMG tests assess the integrity of the large myelinated Aβ fibers, but cannot evaluate the C-fibers or the small-diameter AΔ fibers. Skin biopsies can determine the intraepidermal nerve fiber density (somatic unmyelinated C-fiber termini) and have 90% sensitivity and 97% specificity [48]. In some cases, a nerve biopsy may be appropriate [48]. Regardless of the etiology or clinical course of painful peripheral neuropathy, it has an adverse effect on the quality of life [50].

Gluten neuropathy refers to peripheral neuropathy that is actually the extraintestinal manifestation of gluten sensitivity, defined as having positive gliadin antibodies and/or tissue transglutaminase or endomysium antibodies [51]. In a case-controlled study of 53 patients with gluten neuropathy and 53 matched controls, the gluten neuropathy patients had lower scores in physical functioning, energy, and overall health [51]. In patients with gluten neuropathy, dietary changes may improve both gluten sensitivity and relieve neuropathic symptoms [52]. In a study of 60 patients with gluten neuropathy (77% men, with a mean age of 69.9 years), 55.0% suffered from neuropathic pain. Patients without pain were more likely to be on a strict gluten-free eating plan (55.6% vs. 21.2%, *p*=0.006), and multivariate analysis showed that gluten-free diets were associated with lowering the likelihood of neuropathic pain by 88.7% [53].

In treating peripheral neuropathy, it is important to recognize that pain may occur with any type of neuropathy and should be treated. Neuropathy is so diverse that a single patient may have more than one type of neuropathy. In addition to an accurate diagnosis, risk factors for neuropathy should be addressed and managed, if possible, in order to stop disease progression. Referral to neurologists or pain specialists may be appropriate. Analgesic regimens for peripheral neuropathy are similar to those used for other types of neuropathic pain: multimodal analgesic therapy involving anticonvulsants, antidepressants, opioids, and NSAIDs. Nonpharmacological treatments may be helpful for some patients as well and may be combined with pharmacologic regimens [47].

Management of chronic neuropathic pain

Neuropathic pain was first defined by the International Association for the Study of Pain (IASP) as pain initiated or caused by a primary lesion or dysfunction in the nervous system [2]. This definition was revised in 2008 by the IASP Special Interest Group on Neuropathic Pain and accepted by the IASP in 2011 as “pain caused by a lesion or disease of the somatosensory nervous system” [54,55]. This revised definition eliminated the problematic word “dysfunction,” which would have classified fibromyalgia as neuropathy [54]. The IASP has since adopted the term “nociplastic” to better describe fibromyalgia and other painful conditions [56]. However, an optimal definition of neuropathic pain remains elusive, as lesions of the peripheral or central nervous system may occur in patients with a concurrent neurological dysfunction and pain occurs only in a subset of these patients [54]. In other words, neural lesions do not mean the patient has neuropathic pain [54]. Thus, the presence of a neurological lesion or neurological disease does not guarantee the presence of neuropathic pain, which seems to be more associated with induced changes in the peripheral and central nervous systems, such as alterations of pain modulation systems, central sensitization, and others. The lack of clear definitions has impeded progress in better evaluating, grading, and assessing neuropathic pain [57].

Neuropathic pain can be readily classified as peripheral or central. Peripheral neuropathic pain includes postamputation pain (sometimes called “phantom limb pain”), trigeminal neuralgia, painful radiculopathy, painful polyneuropathy, postherpetic neuralgia (including the optical form [58]), peripheral neuropathy, and peripheral nerve injury pain. Central neuropathic pain includes post-stroke pain, neuropathic pain associated with spinal cord injury, and central pain syndromes involved in MS [57,59,60].

Despite ongoing efforts to better understand the etiology, diagnosis, and treatment of neuropathic pain, many patients do not receive adequate analgesia for their neuropathic pain. The prevalence of neuropathic pain in the general population has been estimated at 7-8%; however, this number must be considered cautiously, as we lack validated diagnostic criteria for use in surveys of the general population [58]. Two large surveys of the general population in the United Kingdom and France estimated neuropathic pain prevalence at 8.2% and 6.9%, respectively [61,62]. The prevalence is substantially higher in certain subpopulations, such as patients with diabetes or cancer.

Most of the epidemiologic data about neuropathic pain come from defined patient cohorts in specific studies. A systematic review of epidemiological studies of neuropathic pain found a prevalence of chronic pain with a neuropathic component to be 3-17% and neuropathic pain associated with a specific disease or condition to vary depending on the condition, estimating the overall population prevalence of neuropathic pain to be between 7% and 10% [63]. The burden of neuropathic pain, including healthcare costs plus lost productivity, is impossible to quantify but is substantial [64].

Numerous guidelines are available for neuropathic pain but are not consistently translated into clinical practice [65]. Guidelines for neuropathic pain care are issued by the IASP [66-68], the European Federation of Neurological Societies [69-71], the National Institute for Health and Care Excellence (NICE), and the Canadian Pain Society [72,73], among others.

Neuropathic pain has been associated with dysfunction, disability, anxiety, depression, sleep disturbances, and reduced quality of life [50,74,75]. The pain of the condition may be substantially influenced by emotional, behavioral, and psychosocial factors. In general, neuropathic pain is associated with overall poor health [76]. Optimal management of neuropathic pain is a clinical necessity, but it goes far beyond just “pain control” [77].

The optimal management of neuropathic pain begins with an accurate diagnosis because there are many different types and manifestations of neuropathic pain. Using the three-L approach to diagnosis (“listen, look, and locate”), the pain site(s) should be identified and pain characteristics described. Pain characteristics may include things such as deep, burning, throbbing, electrical, “pins and needles,” pain qualities as well as whether the pain is intermittent or continuous, if it waxes and wanes, and if it migrates around the body. In addition to the detailed patient history, a physical examination should also be performed. Clinicians must consider the patient’s medical history, underlying conditions, comorbidities, current pharmacological regimen, and what things the patient may have discovered to relieve or exacerbate the neuropathic pain [78]. Early diagnosis improves the likelihood of good outcomes. In treating the neuropathic pain patient, a holistic and patient-centric approach should be used [79]. A key objective in treatment must be pain relief, which may improve physical function, sleep quality, mood, and sense of well-being. In turn, these improvements can boost the quality of life and allow the patient to exercise or move more, which may further improve the physical function [80].

There is general agreement among the numerous guidelines for pharmacologic treatment of neuropathic pain, all of which advise a stepwise approach from the first-line to other treatments [79]. The first-line approach includes tricyclic antidepressants (TCAs) and gabapentinoids [81]. For localized neuropathic pain, some suggest lidocaine patch as the first-line treatment, but it should be noted that anticonvulsants and antidepressants are supported by evidence-based guidelines, whereas the lidocaine patches are supported by data from randomized controlled trials [82]. Pharmacological therapy that affects peripheral sensitization would include capsaicin, local anesthetics, and TCAs. Pharmacological therapy for pain associated with central sensitization would include α2δ ligands (gabapentinoids) [81], TCAs, opioids, and tramadol. In some cases, it is effective to target descending pain modulation using SNRIs, TCAs, opioids, or tramadol [79,81,83,84]. More recently, botulinum toxin has been used to treat certain cases of neuropathic pain; it acts by inhibiting pro-inflammatory mediators and peripheral neurotransmitters from the sensory nerves [85,86]. A treatment algorithm has been published that shows the stepwise progression of various approaches; combination therapy is often appropriate for treating neuropathic pain [80] (Table 1).

**Table 1:** A summary of a treatment algorithm for neuropathic pain showing the stepwise progression of treatments and considerations Source: [79]. NMDA, *N*-methyl-D-aspartate; SSNRI, serotonin-norepinephrine reuptake inhibitor; TCA, tricyclic antidepressant.

Approach	Agents	Considerations
First-line	Tricyclic antidepressants, serotonin-norepinephrine reuptake inhibitors, gabapentinoids. In some cases, topicals, transdermal drugs	Conservative care, multidisciplinary combination, pharmacologic treatment, no opioids
Second-line	Add: Tramadol or tapentadol to the first-line therapy	Combination pharmacologic treatment, tramadol, and tapentadol are atypical opioids
Third-line	Add NMDA antagonists	Interventional therapy may be added
Fourth-line	Neurostimulation	Device-based therapy (combination or monotherapy)
Fifth-line	Low-dose opioids	Do not use >90 morphine equivalent units, opioid-associated side effects may limit treatment
Last-line	Targeted drug delivery using an intrathecal infusion pump system	For refractory pain that cannot be effectively treated with any of the above

The optimal treatment plan should be customized for each patient, considering potential adverse effects, special populations, contraindications, tolerability, drug-drug interactions, and pharmacokinetic profiles. For instance, TCAs may not be appropriate for geriatric patients, particularly at high doses. Opioids should be avoided or used only with careful clinical supervision in patients at risk for substance use disorder [79,80]. Prescribing recommendations for first- and second-line treatments are indicated in Table 2.

**Table 2:** Prescribing recommendations for first-line and second-line treatments for neuropathic pain. The old clinical adage for titration, “start low and go slow,” applies to these agents. Source: [78,79]. SSRI, selective serotonin reuptake inhibitor; TCA, tricyclic antidepressant.

Medication	Starting Dose	Titration	Maximum Dose	Trial Duration
Α_2_δ ligands (gabapentinoids)				
Gabapentin	100-300 mg at bedtime or TID	Increase by 100-300 mg TID every 1-7 days	3600 mg/day	3-8 weeks plus 2 weeks at a maximum dose
Pregabalin	50 mg TID or 75 mg BID	Increase to 300 mg/day after 3-7 days and then by 150 mg/day every 3-7 days	600 mg/day	4 weeks
Selective serotonin reuptake inhibitors				
Duloxetine	30 mg QD	Increase to 60 mg QD after 1 week	60 mg BID	4 weeks
Venlafaxine	37.5 mg QD	Increase by 75 mg each week	225 mg/day	4-6 weeks
Tricyclic antidepressants				
Amitryptiline, desipramine, nortriptyline	25 mg at bedtime	Increase by 25 mg/day every 3-7 days	150 mg/day	6-8 weeks with ≥2 weeks at a maximum tolerated dose
Topical lidocaine 5%				
Topical lidocaine 5%	Maximum three patches/day for 12 hours	None needed	Maximum three patches/day for 12-18 hours maximum	3 weeks

The prescribing choices for the first-line therapy must be carefully considered and customized for each patient [79]. For example, gabapentinoids are recommended as a possible first-line drug class; there are two main drugs with the same mechanism of action to consider: gabapentin and pregabalin. While both are similarly efficacious, pregabalin has a better pharmacokinetic profile, but gabapentin is less expensive. Selective serotonin-norepinephrine reuptake inhibitors (SSNRIs) as well as TCAs are also first-line treatments. In general, TCAs are more effective than SSNRIs, but SSNRIs have a better safety profile. Duloxetine may be a good choice for geriatric patients, as its number needed to harm (NNH) is 17.5 [87]. However, care should be taken in assessing any drug for neuropathy strictly by its NNH or NNT due to heterogeneity among studies [88].

Combination treatments, in which two or more agents with complementary mechanisms of action are used, are familiar analgesic regimens [89,90]. The advantages to combination therapy are several: compared to monotherapy, combination treatment may offer superior analgesia, better tolerability, and minimal common side effects, such as anxiety, depression, and disordered sleep [91]. However, the role of combination therapy for treating chronic neuropathic pain is not particularly well studied. For example, it is not clear as to which, or if, combination(s) of antiepileptics, antidepressants, opioids, topicals, and other agents provide benefits. A systematic review of 21 studies found only limited evidence in support of two-drug combination treatments for neuropathy, mainly because the small number of studies evaluated several different potential combinations [92]. A double-blind study of neuropathy patients treated with either oral nortriptyline, oral morphine, or both in combination found that combination therapy was more efficacious than either agent alone [93]. While combination therapy has numerous advantages, it can also contribute to polypharmacy. Many patients with chronic neuropathy are already taking medications for comorbidities, so combination therapy must be viewed cautiously [94]. In this connection, it is important to avoid prescribing two or more drugs with similar adverse events, such as central nervous system depression [92].

The first step in managing chronic neuropathic pain is to initiate the treatment with one or more of the first-line agents (gabapentinoids, SNRIs, and TCAs) and monitor the patient’s response. If the patient has adequate pain relief and tolerates the medication, then that regimen may continue and the clinical team should monitor the patient to be sure effects are durable. If pain relief is partial, then another first-line medication should be added to see if there is any further improvement. Should the patient not get any pain relief, then the treatment should be discontinued and another first-line treatment used. There can be cases where all first-line options, either as monotherapy or in combination, fail to provide adequate analgesia. In such cases, treatment should advance to second-line approaches (tramadol) and, if those are not effective, then to third-line treatments. In some cases, referral to a pain specialist or neurologist may be appropriate [79,80].

Certain agents have produced equivocal results with respect to control of neuropathic pain. Tapentadol, a µ-opioid receptor agonist, has a mechanism of action that inhibits the descending modulation of pain signals. It is not particularly effective in peripheral neuropathy, although overall it is better tolerated and has fewer side effects than oxycodone [79,80]. In general, oxycodone has shown a degree of effectiveness in painful diabetic neuropathy or postherpetic neuralgia, but not in other painful neuropathic syndromes [95]. On the other hand, tapentadol is effective in treating low back pain with a neuropathic component [96]. Anticonvulsant agents other than α2δ ligands, such as oxcarbazepine, have produced only inconsistent results with respect to neuropathic pain [97]. In recent research, certain oral-mucosal cannabis treatments are effective in treating neuropathic pain, but evidence to date comes from smaller, short-term studies and may not be generalizable to all neuropathic pain patients [98]. Equivocal or mixed results have come from studies of selective serotonin reuptake inhibitors (SSRIs), mexiletine, topical clonidine, and *N*-methyl-D-aspartate (NMDA) antagonists [79,80].

Novel agents in the pipeline for the treatment of neuropathic pain are few. They include subtype-selective sodium-blockers (NaV1.7 antagonists), a novel angiotensin-II antagonist (EMA 401), transient receptor potential vanilloid (TRPV) subtype 1 (TRPV-1) antagonists, and nerve growth factor (NGF) antagonists [79,80].

When evaluating a neuropathic pain patient, it is important to manage the expectations of the patient as well as to be realistic about treatment effectiveness. Complete pain relief is likely an unrealistic goal, and patients should be informed about this fact. A worthwhile clinical goal is >50% pain reduction, but this may not be achievable for all patients. Furthermore, pain regimens should help improve sleep, well-being, function, mood, and quality of life for the patient and must be seen as multidimensional. Pain reduction of 30-50% can be achieved in most neuropathy patients with evidence-guided pharmacologic therapy at maximal doses [99]. Patients should also be advised to consider functional treatment goals as well as just pain relief.

Neuropathic pain secondary to COVID

The symptoms of COVID are many and diverse. Certain prominent symptoms, such as fatigue, anosmia, dysgeusia, headache, vertigo, and myalgia, suggest a direct invasion of the nervous system [100]. Long-haul or long COVID is a recently reported postviral syndrome associated with a constellation of symptoms not necessarily the same as the symptoms the patient experienced with acute COVID [101]. In addition to neuropathic symptoms that may occur during acute or postviral COVID, the treatments to which patients are subjected may further contribute to certain neuropathic pain syndromes. The circulating SARS-CoV-2 virus may increase pro-inflammatory cytokine production (sometimes resulting in “cytokine storm”) and directly invade the olfactory epithelium. Anosmia and ageusia occur with other viral infections but appear to be particularly prevalent among those with acute COVID infections [102]. COVID infection has resulted in an increased rate of neuropathic pain, which in itself is a predictor of neurological complications [100,103].

The pathophysiology of neuropathic pain is described in the literature. Neural lesions can trigger a massive influx of neurotransmitters at the spinal level, leading to intracellular molecular changes, and increase the numbers of certain receivers, such as NMDA, neurokinin-1, aminomethylphosphonic acid, and glutamate along a pathway that results in central sensitization. During a cytokine storm, the release of pro-inflammatory cytokines interleukin-1 (IL-1) and IL-6 increases, which, in turn, increases NGF production. The release of NGF increases localized effects at the Na channels and induces cyclo-oxygenase-2 (COX-2) and prostaglandin production. This promotes depolarization in the form of both antegrade and retrograde axonal transports, resulting in an increase in neuropeptides. The production of neuropeptides can lead to peripheral sensitization. The liberation of tumor necrosis factor (TNF) can also result in anterograde and retrograde axonal transports as well as an increase in the expression of bradykinin receptors and the release of certain neuropeptides, likewise triggering peripheral sensitization. Furthermore, both central and peripheral sensitization can form a feedback loop, intensifying each other [104,105]. In COVID patients, the neuropathic pain mechanisms relate to the host’s response to the virus. Acute COVID infection increases IL-1, IL-6, and TNF-α, all of which stimulate nociceptors [103], and the presence of elevated levels of these particular cytokines have been suspected as associated with the development of neurological symptoms [106].

As with many viral infections, the tissue tropism of COVID requires accessible viral receptors and entry cofactors on the host cells. Neuropilin-1 (NRP1), a transmembrane receptor, appears to enhance the infectivity of the SARS-CoV-2 virus [102]. Thus, NRP1 can be viewed as a host factor for COVID and a potential therapeutic target [107]. There is some preclinical evidence that NRP1 may facilitate the entry of the virus into the brain via the olfactory epithelium [108]. Endothelial dysregulation appears to play a role in severe COVID infection and has been associated with vasoconstriction, vascular leaks, thrombosis, excessive inflammation, and disruption of the body’s natural antiviral immune defenses [109]. It has been speculated that SARS-CoV-2 may bind to the angiotensin-converting enzyme-2 (ACE-2) receptors via the spike protein and, in this way, infect endothelial cells. The suspected downregulation of ACE-2 by the virus may lead to the pulmonary, circulatory, and other complications seen in severe COVID infection [110]. Comorbidities, which seem to exacerbate COVID symptoms, such as obesity and hypertension, involve underlying endothelial damage and dysfunction [111]. Endothelial dysfunction is a systemic condition in which the endothelium no longer promotes vasodilation, fibrinolysis, and anti-aggregation. The healthy endothelium prevents blood clotting by providing an antithrombotic surface, which is disrupted by endothelial inflammation. it appears that COVID infection can cause endothelial dysfunction and a hypercoagulable state [112]. Endothelitis with its hyperproduction of pro-inflammatory cytokines creates a hyperinflammatory state, which can cause the blood-brain barrier to rupture, leading to a cascade reaction of pro-inflammatory mediators and the intrusion of innate immune cells into the brain [113]. Acute COVID infection has been associated with numerous neurological complications, including viral encephalitis, encephalopathy, acute cerebrovascular disease, ischemic stroke, polyneuropathy, epileptic seizures, Guillain-Barré syndrome, and others [114].

Patients who enter intensive care units (ICU) for any reason may be subject to the post-ICU syndrome, a spectrum condition that may involve persistent cognitive deficits, weakness, intrusive memories, and pain [115]. Intensive care in and of itself can be associated with systemic neuropathy; prolonged time spent in the prone position has been associated with neuropraxia and severe axonal damage to the ulnar nerve, brachial plexus, and the nerves in the forearms [116]. It has been estimated that the prevalence of chronic pain one year after ICU discharge ranges from 14% to 77%. Despite this alarming statistic, there has been relatively little study of this population, which expanded greatly during the COVID pandemic [117,118]. Post-ICU syndrome involves chronic painful conditions, such as joint pain, muscle pain related to atrophy, polyneuropathy, and pain associated with the critical illness itself [118].

Much more research is needed to better understand the neurological ramifications of COVID. The healthcare system must anticipate that many COVID survivors will develop de novo neuropathic pain symptoms in the weeks or months following acute infection. COVID survivors who had pre-existing neuropathic painful conditions may experience a deterioration of their condition and exacerbation of their neuropathic pain. The presence of neuropathic pain in a COVID survivor is an indicator of potential neurological damage. Overall, the healthcare system will likely see an increase in neuropathic pain in the coming years [100]. The duration of neurological manifestations of COVID remains unknown.

## Conclusions

Neuropathic pain remains both prevalent and challenging to treat. It often occurs in patients with neurological disorders, such as MS, diabetic peripheral neuropathy, postherpetic neuralgia, spinal cord injury, stroke, or other conditions. Prompt and accurate diagnosis is important for good outcomes, and multimodal pharmacologic regimens are effective. Acute COVID infection as well as the postviral syndrome (“long COVID”) may have neuropathic symptoms that suggest neurological damage.
